# Discovery of the fourth mobile sulfonamide resistance gene

**DOI:** 10.1186/s40168-017-0379-y

**Published:** 2017-12-15

**Authors:** Mohammad Razavi, Nachiket P. Marathe, Michael R. Gillings, Carl-Fredrik Flach, Erik Kristiansson, D. G. Joakim Larsson

**Affiliations:** 1 Centre for Antibiotic Resistance Research (CARe) at University of Gothenburg, Gothenburg, Sweden; 2 Department of Infectious Diseases, Institute of Biomedicine, The Sahlgrenska Academy, University of Gothenburg, Gothenburg, Sweden; 3 Department of Biological Sciences, Genes to Geoscience Research Centre, Macquarie University, Sydney, New South Wales Australia; 4 Department of Mathematical Sciences, Chalmers University of Technology, Gothenburg, Sweden

**Keywords:** Evolution, Bioprospecting, Resistome, Metagenomics, Pharmaceutical, Environment

## Abstract

**Background:**

Over the past 75 years, human pathogens have acquired antibiotic resistance genes (ARGs), often from environmental bacteria. Integrons play a major role in the acquisition of antibiotic resistance genes. We therefore hypothesized that focused exploration of integron gene cassettes from microbial communities could be an efficient way to find novel mobile resistance genes. DNA from polluted Indian river sediments were amplified using three sets of primers targeting class 1 integrons and sequenced by long- and short-read technologies to maintain both accuracy and context.

**Results:**

Up to 89% of identified open reading frames encode known resistance genes, or variations thereof (> 1000). We identified putative novel ARGs to aminoglycosides, beta-lactams, trimethoprim, rifampicin, and chloramphenicol, including several novel OXA variants, providing reduced susceptibility to carbapenems. One dihydropteroate synthase gene, with less than 34% amino acid identity to the three known mobile sulfonamide resistance genes (*sul1–3*), provided complete resistance when expressed in *Escherichia coli*. The mobilized gene, here named *sul4*, is the first mobile sulfonamide resistance gene discovered since 2003. Analyses of adjacent DNA suggest that *sul4* has been decontextualized from a set of chromosomal genes involved in folate synthesis in its original host, likely within the phylum Chloroflexi. The presence of an insertion sequence common region element could provide mobility to the entire integron. Screening of 6489 metagenomic datasets revealed that *sul4* is already widespread in seven countries across Asia and Europe.

**Conclusions:**

Our findings show that exploring integrons from environmental communities with a history of antibiotic exposure can provide an efficient way to find novel, mobile resistance genes. The mobilization of a fourth sulfonamide resistance gene is likely to provide expanded opportunities for sulfonamide resistance to spread, with potential impacts on both human and animal health.

**Electronic supplementary material:**

The online version of this article (10.1186/s40168-017-0379-y) contains supplementary material, which is available to authorized users.

## Background

Bacterial pathogens can become insensitive to antibiotics due to mutations in pre-existing DNA, or by acquisition of antibiotic resistance genes (ARGs), many of which are likely to originate from environmental bacteria [1]. These genes spread via mobile genetic elements, such as plasmids and transposons, which facilitate the transfer of genetic material between bacterial cells and species [2]. Integrons play a major role in the acquisition and dissemination of ARGs. These genetic elements capture and express genes; they are often associated with transposons and can be carried by conjugative plasmids [3]. Integrons are composed of three key features: an integron integrase gene (*intI*), an integron-associated recombination site (*attI*), and an integron-associated promoter (Pc). The *intI* gene encodes a site-specific tyrosine recombinase, which performs integration and excision of genetic elements, known as gene cassettes, at the recombination site, *attI*. Then, the integrated gene or genes are expressed by a dedicated promoter (Pc) embedded in *intI* or the *attI* site. This mechanism for the integration and excision of new functional modules helps bacteria rapidly adapt to selection pressures, including the acquisition of resistance phenotypes [4].

Integrons have been found in ~ 6% of all sequenced bacterial genomes [5]. These ancient genetic elements can recruit diverse gene cassettes, most often encoding proteins of unknown function [3]. In contrast, integrons carried by pathogens are often resident on mobile elements and carry resistance genes. The relative abundance of mobile integrons in pathogens, particularly the class 1 integron, is now much higher than it is in environmental organisms [6–10]. The success of these clinical class 1 integrons depends partly on their association with transposons of the *Tn*402 and *Tn*21 families. In particular, the *Tn*402 transposon targets the resolution (*res*) site of plasmids, thus inserting the class 1 integron into a wide diversity of conjugative plasmids [3].

Genes encoding resistance to almost all families of antibiotics have been accumulated by class 1 integrons over the last 100 years [11, 12]. The recruitment of novel resistance genes into integrons is thus of considerable concern [13, 14], and their presence in non-pathogenic species or environmental microbial communities implies a risk for future transfer to human pathogens. Given the vast diversity of both bacteria and genes in the external environment [3, 15], environmental gene cassettes are likely to be an important source of novel resistance genes to pathogens.

Early knowledge of genes with a potential to become clinically relevant resistance genes is important, because this helps us to better understand how resistance develops and to prepare surveillance and control measures to reduce their dissemination. Both functional metagenomics and sequence-based metagenomics have been used in the past to identify candidate resistance genes in environmental communities [16, 17]. Functional metagenomics relies on phenotypic screening and is thus hampered by the high abundance of well-characterized resistance genes. This makes functional metagenomics cost- and labor-intensive to find rare, novel resistance genes. Sequence-based metagenomics relies on similarities to known resistance genes, thus easily missing truly novel genes. Additionally, the overwhelming majority of the sequenced DNA has no relevance for resistance, increasing sequencing costs and computation [18]. Neither of these techniques can easily pinpoint mobile genes that have a higher probability of being transferred to pathogens. An approach that specifically targets mobile resistance genes would have the potential to identify genes of concern in a more efficient way. Novel ARGs located in mobile elements, such as clinical class 1 integrons, would be at increased risk of becoming a clinical problem [13].

In addition to focusing on mobile elements, exploring microbial communities with existing selection pressure from antibiotics would probably further increase the chances of finding novel resistance genes. Environments impacted by discharges from antibiotic manufacturing could hence be relevant to investigate. For about a decade, we have studied an Indian treatment plant that receives highly antibiotic-contaminated wastewater from drug manufacturing. As much as 80% of the bacteria isolated from this environment harbor class 1 integrons [19]. Downstream river sediments also contain elevated abundances of class 1 integrons [20]. The antibiotic consumption in India is high and to a large extent uncontrolled [21]. Accordingly, antibiotic resistance in, for example, *Enterobacteriaceae* has become a major problem [22, 23]. Environments where untreated sewage is mixed with “environmental” bacteria could therefore be worthwhile to explore for novel resistance genes.

In this study, we have characterized class 1 integrons to identify novel ARGs and to expand our knowledge of the gene cassettes employed in integrons. Amplicons of integron gene cassettes were generated from Indian river sediments that were heavily contaminated by industrial discharges of antibiotics and by untreated sewage and hospital waste [19, 24].

## Results

Sequencing amplicons resulted in 216,807 long PacBio reads (LRs or partial integrons) with an average length of 1.25 kilobases (kb) and 14,184,598 short Illumina reads (SRs) with a maximum length of 250 bases. After filtering low-quality reads, 13,506,840 SRs along with all the LRs were fed into Proovread [25], resulting in 170,257 corrected LRs. Clustering of the LR dataset resulted in 102,550 non-redundant reads (Table 1). A total of 198,436 open reading frames (ORFs) were identified by Prodigal [26]. Clustering of all identified ORFs at 99% amino acid identity led to 19,723 unique ORFs. The ORFs were annotated against the NCBI protein and nucleotide database to identify their putative function. To the best of our knowledge, the numbers of different partial integrons and putative gene cassettes in this study are considerably higher than any previous study that has identified gene cassettes from environmental samples (Table 2).

**Table 1:** Total number of unique long reads by sample and primer combinations in the sequenced amplicons

		Primers			
		HS464-GCP2	HS458-HS459	MRG284-MRG285	Primers not detected
Samples	RS_PETL_	30,780 (1.3 kb)	11,317 (1.2 kb)	6665 (1.1 kb)	594 (1.1 kb)
	RS_Pune_	25,229 (1.4 kb)	16,182 (1.3 kb)	10,853 (1.1 kb)	930 (1.1 kb)

The average lengths of the long reads are presented in parenthesis

**Table 2:** Comparison with previous PCR amplicon studies of gene cassettes

Study	Sample type	Location	No. of unique ORFs
[43]	Marine sediment	Halifax Nova Scotia, Canada	1372
[44]	Sludge	Sydney Tar Ponds, Nova Scotia, Canada	708
[70]	Sediment	Minas Gerais state, Brazil	143
[42]	Marine sediment	Suez and Tokyo Bay	146 + 68
Current study	Sediment	Pune and Patancheru, India	19,723

**Fig. 1:**
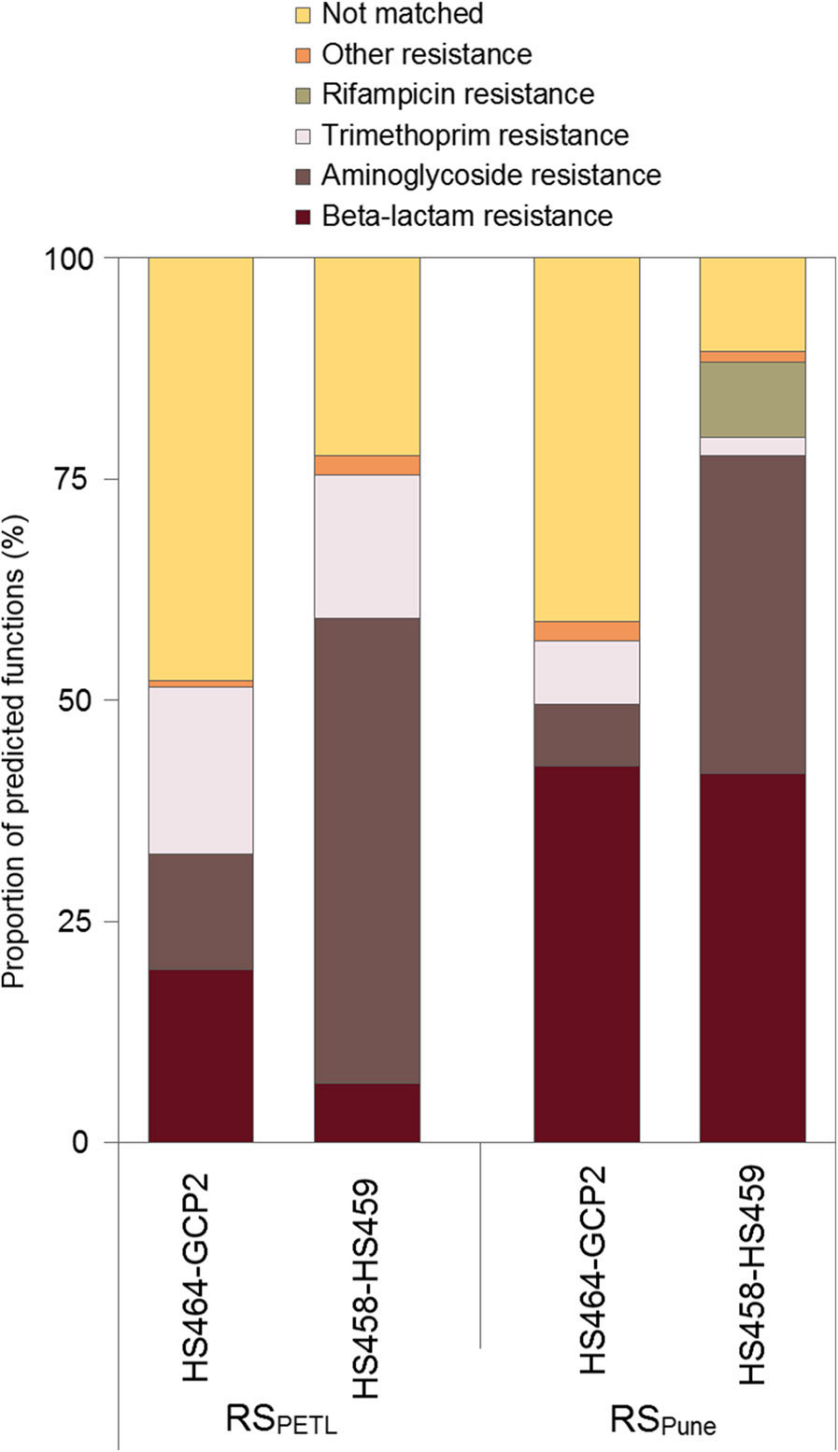
Predicted functions of open reading frames separated by samples and primers. The results are based on known homologues in the CARD database

For both sites, resistance gene cassettes (known and putative) dominated (51 to 89%) (Fig. 1 and Additional file 1: Figure S1). The primer pair HS458-HS459, which was designed to preferentially recover “clinical” integrons, showed the strongest dominance of known and putative resistance gene cassettes (78 to 89%). The primer pair HS464-GCP2, which recovers a slightly more even mix of clinical as well as environmental (pre-clinical) integrons, contained a somewhat lower proportion (52 to 59%), whereas 51 to 73% of the amplicons from the MRG284-MRG285 primer pair included known and putative resistance gene cassettes (Additional file 1: Figure S1). The latter pair recovers primarily chromosomal integrons. Additional file 1: Figure S2 illustrates putative functions of the ORFs of gene cassettes in the “clinical” versus “environmental” integrons from the HS464-GCP2 primer pair (see the Methods section). The distribution of putative functions was very similar between the two.

Known and putative ARGs are reported in Additional files 2 and 3. The number of genes providing resistance to different classes of antibiotics is indicated in Table 3. Moreover, to assess if the identified ORFs included genes that were not previously described as integron gene cassettes, we matched them against INTEGRALL [27]. Of the 19,723 non-redundant ORFs identified in this study, 5942 (~ 30%) were previously reported (see the Methods section). However, the rest of the ORFs had lower nucleotide similarity (identity < 95% and coverage < 70) to the sequences deposited in INTEGRALL. Additional file 1: Figure S3 shows the putative function of these recent ORFs based on known homologs in the NCBI protein database. Hypothetical proteins form the largest portion in both samples, followed by ARGs (known as well as putatively novel). The full list of these ORFs, annotated based on NCBI protein database, is presented in Additional file 4.

**Table 3:** Number of known and putative novel ARGs to different families of antibiotics

Antibiotic family	Number of known ARG	Number of putative novel ARGs
Aminoglycoside	32	689
Beta-lactam	44	103
Trimethoprim	8	240
Rifampicin	3	2
Chloramphenicol	8	4
Macrolide	3	7
Sulfonamide	2	1
Quinolone	1	0

**Fig. 2:**
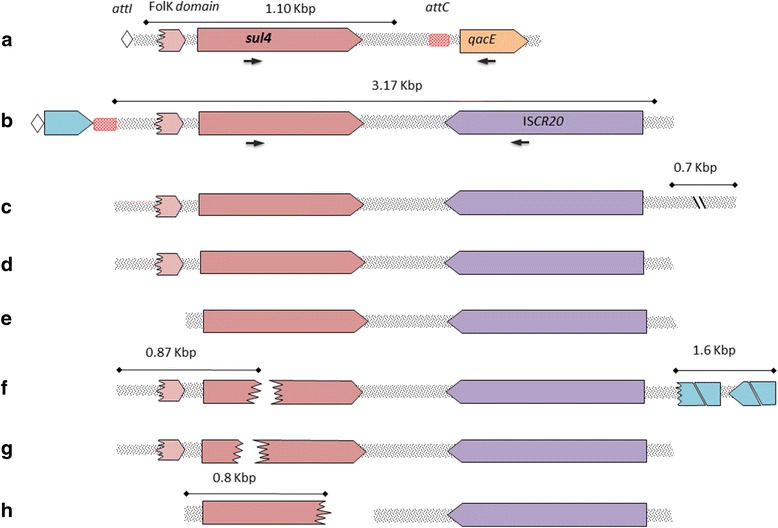
Schematic diagram of the different arrangements of *sul4* recovered by amplicon sequencing of integron datasets: **a** RS_PETL_, Hyderabad, India. The read begins with an *attI* site, as the integron-integrase gene was not amplified by the primers. Alignment of 1.10 Kbp of the gene cassettes to the read in **b** RS_Pune_, Pune and RS_PETL_, Hyderabad, India. The first gene cassette is a hypothetical protein (WP_019224580.1). Primers used to further confirm the context found in RS_Pune_ and RS_PETL_ are indicated by arrows. The rest of the arrangements are contigs that resulted from assembling different shotgun metagenomics datasets. **c** mgm4622354.3 (MG-RAST ID) collected from Kolkata, India. **d** mgm4510219.3 collected from Malaysia. **e** mgm4709385.3 collected from Sheffield, UK. **f** ERR1414268 (EBI ID) collected from Käppala sewage treetment plant, Sweden. Downstream of IS*CR20* are hypothetical proteins (WP_076836759.1 and WP_011927925.1) **g** mgm4537907.3 collected from Hong Kong and **h** mgm4714564.3 collected from Beijing, China. Due to the lower read coverage, contigs covering the entire *sul4* gene were not recovered in the **f**–**h** metagenomes

Nine of the novel genes predicted to provide resistance to aminoglycosides, beta-lactams, rifampicin, chloramphenicol, trimethoprim, and sulfonamides were tested and functionally confirmed by expressing them in *Escherichia coli* (Additional file 1: Table S1). These genes and their contexts are presented in Fig. 2 and Additional file 1: Figure S4. Phylogenetic analysis of all putative OXA-variants showed that their closest relatives were OXA-2, OXA-10, and OXA-46 (Additional file 1: Figure S5). The identified OXA-10 variants did not contain the N143S and G157D substitutions, which are associated with ceftazidime resistance and extended spectrum beta-lactamase resistance (ESBL) characteristics [28]. When expressed in *E*. *coli*, all provided resistance to ampicillin, whereas the synthesized OXA-10-like gene also conferred resistance to cefotaxime. All the tested OXA variants conferred reduced susceptibility to carbapenems corresponding to a 21- to 63-fold increase in the minimal inhibitory concentration (MIC) for ertapenem and around a two- to three- fold increase for imipenem.

A putative sulfonamide resistance gene with 69% amino acid identity to the closest known dihydropteroate synthase (DHPS) and between 31 and 33% identity to known mobile sulfonamide resistance genes (Table 4) was identified. Experimental validation showed that this gene conferred full resistance to sulfamethoxazole with an MIC > 1024 μg/ml (more than 256-fold increase compared to control). Based on its ability to provide sulfonamide resistance, its mobile character, as demonstrated by its presence in integrons, and the homology to previously known sulfonamide resistance genes, we proposed the name *sul4*(GenBank: MG649393). To date, only three different mobile sulfonamide resistance genes have been identified, whereas for most of the other classes of antibiotics, many more resistance genes are known (e.g., for beta-lactams, aminoglycosides, tetracyclines, and trimethoprim). Since a new mobile sulfonamide resistance gene is a significant observation, we focused on this gene for further characterization.

**Table 4:** Similarity of the Sul4 protein to the closest known dihydropteroate synthase and the three previously known mobile sulfonamide resistance proteins

Accession in NCBI	Identity (%)	Coverage (%)	Description	Year of discovery
CUS02277.2	69	99	Dihydropteroate synthase [Ardenticatena]	2016
AEJ33969.1	32	92	Sul1	1975
AAL59753.1	33	80	Sul2	1988
ACJ63260.1	31	93	Sul3	2003

Fourteen LRs in river sediment collected in Pune (RS_pune_) and 48 LRs in river sediment collected near PETL (RS_PETL_) contained *sul4*. These represent two different cassette arrangements. In the first case, *sul4* was the first cassette after the *attI* site, followed by the complete *qacE* gene, and found only in RS_PETL_ (Fig. 2a). In the second case, found in both samples, *sul4* was the second gene cassette following a hypothetical protein, which then was followed by a transposase from the insertion sequence common regions (IS*CR*) family (Fig. 2b). These two arrangements were further confirmed by performing PCR on the original unamplified samples using primer pairs targeting *sul4* and *qacE*/IS*CR20* (Fig. 2a, b), both of which generated amplicons of the expected size.

Screening 6489 publicly available metagenomic datasets revealed the presence of *sul4* in seven different countries across Asia and Europe (Table 5). The detailed descriptions of these datasets along with normalized counts of sulfonamide resistance genes are presented in Additional file 5. Figure 2c–h shows the recovered contigs containing *sul4* from some of these datasets. The full list of datasets examined is presented in Additional file 6.

**Table 5:** Summary of 108 shotgun metagenomics datasets containing reads which are mapped to Sul4 with 100% identity, covering more than 20 amino acids (see Additional file 5)

Country	No. dataset	No. mapped reads	Biome	Collection date
India	38	10,803	Aquatic	2008–2016
China	21	249	Air,terrestrial,aquatic	2011–2016
Hong Kong	1	34	Aquatic (sewage)	2013
Malaysia	1	15	Mangrove	2011
Sweden	32	422	Aquatic (sewage)	2012
UK	9	290	Aquatic (sewage)	2016
Switzerland	6	46	Aquatic (sewage)	2014

**Fig. 3:**
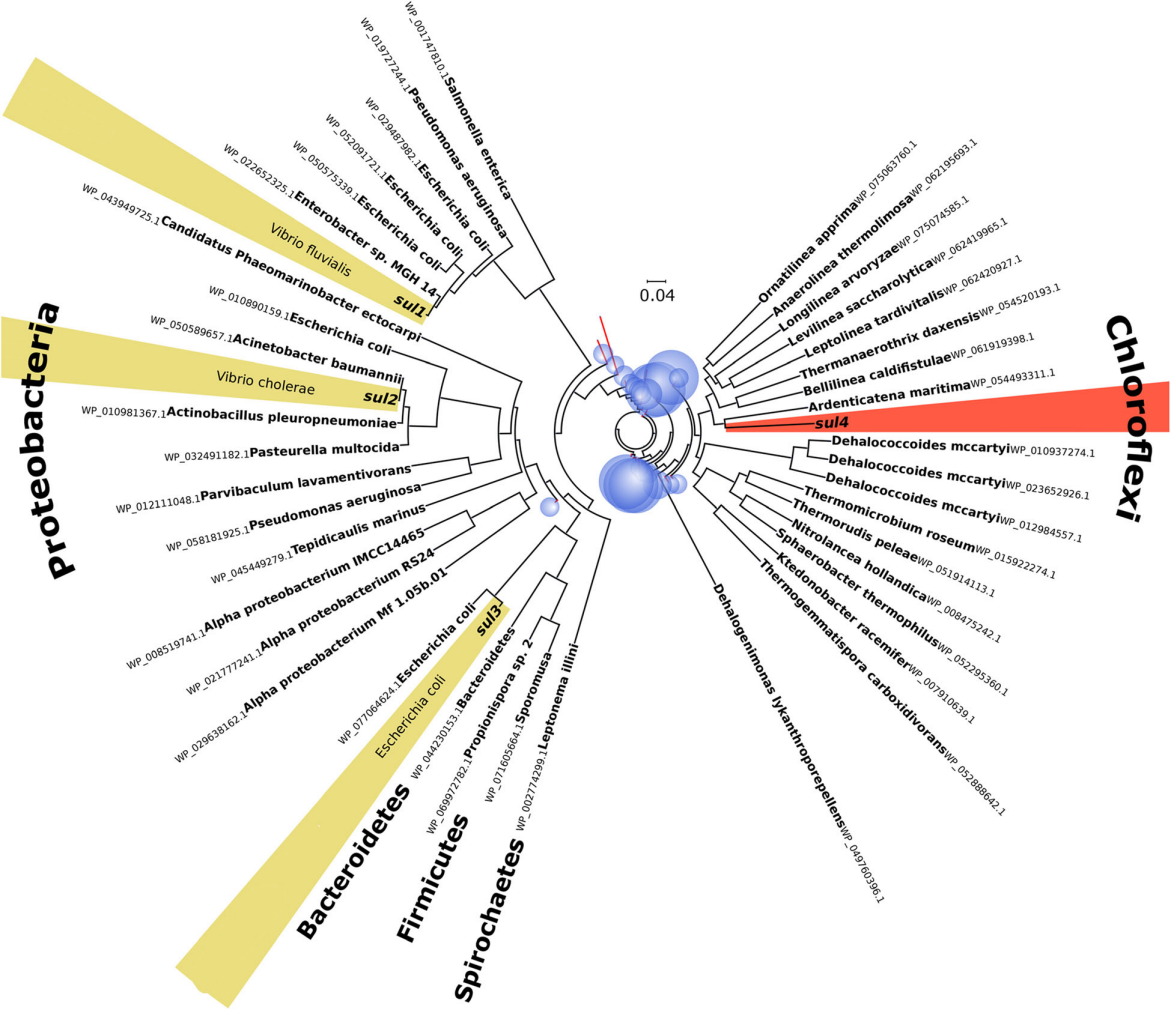
Collapsed phylogenetic tree of Sul4 and known chromosomal dihydropteroate synthase proteins from the NCBI RefSeq database excluding plasmid-born genes, along with proteins encoded by mobile sulfonamide resistance genes *sul1*, *sul2*, and *sul3* from the CARD database. Branches are annotated with the accession number and the identified species name, with the phyla in bold. Collapsed clades are distinguished by red edges and the size of the blue bubbles corresponds to the number of proteins in the collapsed clade. The full version of the tree is available as an Additional file 9 in Newick format

The collapsed phylogenetic tree of Sul4 and 8875 different dihydropteroate synthase enzymes (protein similarity less than 95%) retrieved from the NCBI protein database are presented in Fig. 3. The Sul4 protein was located in a clade with DHPS proteins from members of the phylum Chloroflexi, found in various environments, including wastewater [29].

A structural prediction of Sul4 and the other three sulfonamide resistance proteins is presented in Additional file 1: Figure S6. All the proteins are structurally similar due to the preservation of the distorted cylinder in the center, with α-helices around the inner β-strands and coils. Additional file 1: Figure S7 also presents the alignment and the comparison of the secondary structures between Sul1, Sul2, Sul3, and Sul4 and a sensitive DHPS (with protein data bank (PDB) ID: 1AJ0) [30].

## Discussion

We used a targeted PCR of integrons from polluted environmental samples followed by amplicon sequencing using next generation sequencing technologies to greatly extend our knowledge of mobile antibiotic resistance genes found as gene cassettes. Combining the accuracy of short reads from Illumina sequencing with the higher resolution of long reads from PacBio sequencing generated a clear view of the gene cassettes and their immediate context, also providing clues about their evolutionary history. We therefore suggest that a similar approach could be applied to search for ARGs as well as other functional genes in different contexts.

Putative novel ARGs for aminoglycosides, beta-lactams, trimethoprim, rifampicin, chloramphenicol, and sulfonamides were identified. Several novel OXA variants provided reduced susceptibility to carbapenems, providing an additional battery of integron-borne genes that could contribute to resistance against last-line antibiotics. Many of the known and putative ARGs that were found were previously not reported as gene cassettes, thus revealing a potential to be spread via integrons.

A mobile sulfonamide resistance gene with only 31–33% identity to previously known mobile sulfonamide resistance genes was discovered, providing a very high level of resistance when expressed in *E*. *coli*. Only three mobile sulfonamide resistance genes (*sul1*, *sul2*, and *sul3*) have previously been identified. The *sul4* gene was retrieved by amplifying gene cassettes using class 1 integron-specific primers, the most common type of integron found in human pathogens. The gene cassette contains an ORF for the *sul4* gene, and a partial domain of FolK (COG0801) that is present in the upstream region of *sul4*, similar to the fused *folKP* gene in chlamydia. This domain can be found in dihydroneopterin aldolase (i.e., FolK/SulD) which is involved in the folate biosynthesis pathway. The enzyme encoded by *folK* harbors the activity of EC 4.1.2.25 and produces 6-hydroxymethyl-7,8-dihydropterin diphosphate, which later is used by the dihydropteroate synthase (EC 2.5.1.15) [31]. Downstream of *sul4*, a transposase (IS*CR20*) belonging to the IS*CR* family is identified in one of the two contexts. This family of insertion sequences lacks inverted repeats (IR) and, without the need of another transposase protein, they can be mobilized along with their adjacent DNA sequence through rolling-circle (RC) transposition [32, 33]. We did not find any other ARGs adjacent to IS*CR20* in the studied samples; however, IS*CR*s, such as IS*CR1*, have been found adjacent to the 3′ conserved segment (3′-CS) of integrons, and with the loss of their *terIS* sites, they can mobilize the entire integron. Moreover, sulfonamide resistance genes are also known to be carried by IS*CR* elements (e.g., association of *sul2* and IS*CR2*, GenBank: KX900483.1) and are reported in complex integrons (e.g., *sul1* in GenBank:AY079169.1). The gene encoding the IS*CR20*-like protein, found in the integron near *sul4*, has been reported earlier to be adjacent to *sul2* in the *Bibersteinia trehalosi* genome (GenBank:CP006956.1_(295,195..297771)_) and as a complex integron in *Enterobacteriaceae* isolates (GenBank:DQ520941.1_(1873..3163)_). Taken together, these findings provide strong support that *sul4* has been decontextualized from the chromosome of its original host. The IS*CR* could potentially provide mobility to the entire integron.

Structural prediction of Sul4 indicates strong overall similarities to Sul1, Sul2, and Sul3. The α/β barrel structure contains the binding sites for 7,8-dihydropterin pyrophosphate (DHPP), para-aminobenzoic acid (pABA), and sulfonamide. After DHPP has bound deep in the cylinder, sulfonamide binds near the surface of the protein. Thus, sulfonamide binding is affected by changes near the surface (e.g., insertion of amino acid in coils after amino acid 190) of DHPS [34]. Most of the α-helices in Sul4 are preserved, but the coils and β-strands have changed considerably from sensitive DHPS, which possibly contributes to reducing the affinity of sulfonamide and the Sul4-DHPP complex structure.

Although extensively used since 1935, the use of sulfonamides in human medicine has become mainly limited to treating gastrointestinal or urinary infections. However, sulfonamides are still broadly used in animals for treatment, growth promotion, and prophylactic purposes. There is a lack of reliable records for the global usage of sulfonamides in animals. Data covering 10 European countries show that sulfonamides and trimethoprim constitute 17% of the sales of veterinary antibacterial agents [35], and in the US, 380,186 kg of sulfonamides was distributed legally during 2015 for food-producing animals [36]. High concentrations of sulfonamide residues in animal manure in China indirectly indicate heavy usage [37, 38]. Hence, further spread of sulfonamide resistance would have severe consequences, particularly for the animal sector.

Fourteen years has passed since the discovery of the third mobile sulfonamide resistance gene. The fact that so few genes have been detected, despite almost 80 years of intense usage of sulfonamides, is intriguing, as there are considerably more types of mobile genes for tetracycline resistance, beta-lactamases, or aminoglycoside acetyltransferases [39]. Our finding of a fourth mobile sulfonamide resistance gene indicates that there are still ongoing forces that introduce, mobilize, and maintain new sulfonamide resistance genes in bacterial communities. We do not yet know the present host-range of the *sul4* gene, nor its context outside of integrons. Our results show, however, that *sul4* provides high-level resistance in *Escherichia coli*. This finding suggests that *sul4* can provide clinical resistance in *Enterobacteriaceae*, similar to the previously discovered sulfonamide resistance genes. Because of founder effects [40], one may question how effectively *sul*4 might spread. It might be that the founder effect is not critical, as is apparent from the spread of beta-lactamases and other types of resistance genes. Moreover, the presence of *sul4* in different samples from different continents suggests that the gene has found a way to spread successfully.

The *sul4* gene was abundant both at the PETL and Pune sites (Fig. 2b). The recovered contigs from Sweden and Kolkata suggest that IS*CR20* has had a role in mobilizing *sul4* and its flanking regions, probably via rolling-circle transposition. In Sweden, the partially recovered *sul4* and the IS*CR20* were located upstream from two hypothetical proteins. It seems that the IS*CR20* has truncated one of the hypothetical proteins, as we could not find the full length ORF (Fig. 2f). In the Kolkata samples, the *sul4* and IS*CR20* were adjacent to an unknown sequence with no detectable ORF. These downstream sequences, which do not appear to follow the structure of a classical integron, suggest the insertion of *sul4* and flanking regions in different regions of the bacterial host genomes. In China, *sul4* was found in Beijing smog in three different samples, and highlights the possible role of aerial transport of this ARG. Unfortunately, these datasets are not sequenced deep enough to assemble the reads and investigate the context of *sul4*.

An association of *sul4* with the phylum Chloroflexi, as suggested by phylogenetic analysis, is further supported by the high abundance of *sul4* in aquatic metagenomes from an algal bloom in Kolkata. No reads of this datasets were mapped to other mobile sulfonamide resistance genes, which are typically markers of anthropogenic pollution. Studies have shown that the phylum Chloroflexi is one of the dominant bacterial phyla in these aquatic ecosystems [41]. We believe that further investigations on Chloroflexi could provide clues about the original host of *sul4* and how it has been decontextualized.

Amplifying integrons from polluted river sediment resulted in identification of a large range of gene cassettes, the majority of which were known or putative ARGs. To our knowledge, such high diversity of ARGs in integron gene cassettes has not been described previously in any bacterial community [42–44]. Prior selection by antibiotics is the most plausible explanation behind the selection of bacteria with such cassettes, either in the actual sediment and/or in the gut microbiota of humans that contribute fecal residues to the sediment. Selection by antibiotics is likely an important factor in the initial mobilization of such genes, enabling them to shift from a functional role in general metabolism to become mobile resistance genes. The high abundance of resistance gene cassettes both in environmental and clinical integrons indicates an extensive exchange of gene cassettes between them. Close interactions between different types of integrons could facilitate the accumulation of novel resistance determinants and virulence factors into clinical integrons. Moreover, the finding of DNA from human fecal bacteria together with a high abundance of integrons at both sampling sites [20, 24] further highlights the opportunity for such interactions potentially allowing a gene flow of novel resistance determinants to pathogens. Therefore, these results provide part of the necessary ecological connectivity that could contribute to increased resistance in clinics [14, 45].

## Conclusions

A targeted amplicon sequencing approach was used to greatly extend our knowledge of integron-born gene cassettes, particularly those with antibiotic resistance function. Combining the accuracy of short reads with the higher resolution of long reads generated a clear view of the gene cassettes and their immediate context, providing some clues about their evolutionary history. A range of novel resistance gene cassettes against different families of antibiotics were identified, including the fourth mobile sulfonamide resistance ever found, namely, *sul4*.

## Methods

### Sample collection

Sediments from the Mutha River (RS_Pune_) were collected from within the city of Pune in Maharashtra, India, (referred as Pune river or RS_Pune_) and pooled into one composite sample (for details, see Additional file 1: Table S2). Pune is the second largest city in the state of Maharashtra, and the river passing through the city is heavily contaminated by untreated sewage [24]. Sediment samples were also collected from the Isakavagu/Nakkavagu River, which flows past an industrial waste water treatment plant (Patancheru Enviro Tech Ltd.; PETL) near Hyderabad, India. The PETL samples, described previously [20], were pooled and are referred to here as RS_PETL_. The treated waste water and river sediments were contaminated with exceptional levels of fluoroquinolone antibiotics (up to 31 mg/L and up to 0.9 g/kg organic material, respectively) and harbor bacterial communities with very a high abundance of resistance genes and integrons [15, 19, 20, 46, 47].

### DNA extraction, PCR, and sequencing

**Fig. 4:**
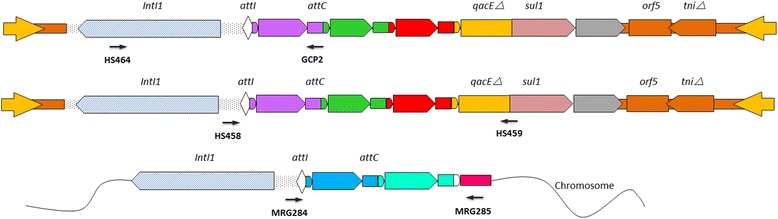
Map of primer pairs used to amplify partial class 1 integrons

Total genomic DNA was extracted from individual frozen sediment samples using the PowerSoil® DNA isolation kit (MoBio, Carlsbad, CA) according to the manufacturer’s instructions (note that unamplified DNA was used, in contrast to repliG-amplification as in [20]). Concentration of the extracted DNA was determined using a dsDNA High Sensitivity (HS) Assay kit on the Qubit® Fluorometer (Invitrogen, USA). The subsamples were pooled, and DNA from each sample was amplified using three sets of previously used primer pairs (Fig. 4) [42, 48, 49]. All PCR reactions were carried out using phusion high-fidelity DNA polymerase (Thermo Scientific, USA). The primers HS458-HS459 amplify entire gene cassette arrays by binding to the 5′ and 3′ conserved segments of clinical class 1 integrons. The primers HS464-GCP2 target the class 1 integrase gene and a conserved region of the *attC* recombination site. The primers MRG284-MRG285 amplify the entire gene cassette array from the *attI* site to a conserved region beyond the cassette array in the pre-clinical class 1 integrons; PCR products were purified using a PCR purification kit (Qiagen, Germany) and quantified using the Qubit® Fluorometer. Amplicons were then sent for single-molecule real-time (SMRT) sequencing technology (Pacific Biosciences) to LRs and shotgun metagenomic sequencing to produce SRs (paired-end 250 bp reads on the Illumina Mi-Seq2000 platform) at Science for Life Laboratories (Uppsala and Stockholm, Sweden). The library construction of LRs was carried out using the SMRTbell Template Prep Kit 1.0 (part number: 100-259-100). The SMRT-bell libraries were sequenced on a PacBio RSII platform with P5-C3 chemistry using two SMRT cells. The metagenomic sequencing data and the corresponding meta-data have been deposited in the NCBI database under the Bio-Project ID: PRJNA400874.

### Sequence analysis

The quality of the SR dataset was assessed using FastQC [50]. Reads with low-quality bases were trimmed to reach a score of 21, and those with less than 80 bases in length were filtered using high-throughput quality control (HTQC) [51]. If only one end of the paired-end reads had acceptable quality, we used it as a single read. The resulting paired and single reads were used to correct LRs with Proovread as a hybrid correction pipeline for single-molecule real-time sequencing [25]. Proovread maps SRs to LRs using sequence alignment and then, with the generated short-read consensus, corrects errors in the LRs. Proovread also calculates updated position-specific quality scores based on the coverage and composition of the consensus.

Redundant LRs were identified by clustering them using CD-HIT (with following parameters: -c 1 -uS 0.05 -S 5 -n 8 -d 0 -r 1) [52]. Blastn in the BLAST+ package (with following parameters: mode blastn-short, word_size 7, gapOpen 5, gapextent 2, reward 1, penalty -3) was employed to find the primers in the 5′ and 3′ ends of the LRs [53]. The LRs were annotated as follows. First, the ORFs were predicted using Prodigal (-p meta) [26]. The functions of the ORFs were identified through similarity searches against the non-redundant nucleotide and protein NCBI databases (update at 07 November 2016). BLAST+ in blastn mode was used for the nucleotide alignments while Diamond was used for the protein alignments. ORFs which were not annotated as integrase or with related terms (i.e., *IntI*, *IntI*1 integrase, XerD domain) or *qacEΔ* or related terms (i.e., qacE delta, partial quaternary ammonium compound resistance protein, partial ethidium bromide resistance protein) and were longer than 75 amino acids were considered putative integron gene cassettes. The LRs amplified by HS464-GCP2 primers were further divided into “clinical” and “environmental” integrons [54]. Blastn in the BLAST+ package with a sequence identity of 100 was used to identify a previously widespread clinical integrase (NCBI accession ID: KC417379_(1..1014)_) from downstream of the HS464 primer sequence. The rest of the LRs were classified as environmental integrons.

Putative novel resistance genes were identified based on their sequence identity and the length of the alignment (coverage) to known homologues in CARD (version 1.1.0) [55] and the NCBI database. We classified ORFs with at least 95% identity to closest homologs in the CARD database as “known resistance genes” and those with identity between 60 and 95% and with coverage greater than 65% as “putative novel resistance genes.” The gene cassettes with known function were clustered to remove redundancy using CD-HIT. HattCI was used to identify *attC* sites in the LR [56].

The abundance of mobile sulfonamide resistance genes (*sul1*, *sul2*, *sul3*, and *sul4*) was quantified in 6489 metagenomic datasets as follows. First, shotgun metagenome datasets were collected from the MG-RAST database [57] (sequence type: shotgun metagenome) and our local database from previous studies. The reads were mapped to the Sul proteins using Usearch (with following parameters: -search_global -id 1 -maxaccepts 0 -maxrejects 0) [58], and the best hits with higher sequence identity and longer alignment length were selected. Datasets containing more than five reads mapped to Sul4 were analysed with Metaxa 2.1 [59] to extract the number of bacterial 16S rRNA sequences (SSU). The count data were normalized as was done in a previous publication [15]. To identify the context of *sul4*, the reads in the selected metagenomic datasets were filtered and trimmed using HTQC and assembled using Megahit 1.1.1 [60].

The phylogenetic analysis on the *sul4* and OXA variant gene cassettes was done as follows. Chromosomal proteins annotated with the term “dihydropteroate synthase” were retrieved from the NCBI RefSeq database. All 18,822 proteins along with the Sul1, Sul2, and Sul3 proteins from the CARD database were clustered using CD-hit (with parameters -c 0.95) to remove redundancy. Beta-lactamases classified as OXA were also retrieved from the CARD database. Multiple alignments were done using MAFFT (--auto) [61], which brought efficiency to the pipeline with its parallelism implementation and efficient memory utilization. Then, phylogenetic trees were produced by quicktree [62] using the neighbor-joining algorithm. The Python package ETE3 was used to draw and collapse the phylogenetic trees [63] to better visualize the relationship between the mobile sulfonamide resistance genes and their closest relatives.

To identify genes previously not described in integrons, LRs were searched against the INTEGRALL database [27]. All the accession numbers (*n* = 8471 November 2016) in INTEGRALL were retrieved, and their sequences were downloaded from the NCBI GenBank database. The collected sequences were utilized as a reference database for nucleotide comparison between the LRs and recorded ORFs, using blastn in the BLAST+ package. Novel ORFs in the integrons were identified based on the sequence identity and the length of the alignment. We classified hits with an identity greater than 95% and coverage greater than 70% as previously reported integron-associated ORFs.

### Functional verification of candidate novel resistance genes

Putative novel resistance genes were grouped according to the classes of antibiotics against which they were likely to confer resistance. Nine candidate novel genes/gene variants with high correction scores and low identity to the closest known resistance gene in each class were selected for functional verification. The candidate novel genes were synthesized at ThermoFisher Scientific, Germany, using their GeneArt Gene Synthesis service and subcloned into the expression vector pZE21-MCS1 using *Kpn*1 and *Bam*H1, as described previously [64]. The recombinant plasmids containing novel resistance gene candidates were then transformed into *E*. *coli* C600Z1 (Expressys, Germany) by electroporation. The MICs of the antibiotics for the strains containing the candidate novel resistance genes were determined using E-tests on Mueller-Hinton Agar plates (BioMérieux, France) with the addition of 100 ng/μl anhydrotetracycline (aTC), which acts as an expression inducer for the pZE21-MCS1 gene inserts [65]. The strain containing empty vector was used as a negative control. The protein sequences of the synthesized genes are presented in Additional file 7.

## Additional files

Additional file 1: **Figure S1.** Predicted functions of open reading frames recovered by the chromosomal integron primer pair MRG284-MRG285 separated by samples. The results are based on known homologues in the CARD database. **Figure S2.** Predicted functions of open reading frames of the “clinical” and “environmental” integrons from the HS464-GCP2 amplicons separated by samples. The results are based on known homologues in the CARD database. **Figure S3.** Functional annotation of the open reading frames not previously reported in integrons. The results are based on known homologues in the NCBI protein database. Putative resistance genes are determined based on annotation in the NCBI database. **Figure S4.** Genetic arrangements of functionally verified resistance gene cassettes as identified by PCR amplification of the integrons. The synthesized gene cassettes are distinguished by thicker borders. Both synthesized OXA-2-like gene cassettes have the same arrangement. **Figure S5.** Collapsed phylogenetic tree of the identified OXA-variant gene cassettes and 289 known OXA-variants retrieved from the CARD database. The identified genes are described by Id numbers and located adjacent to OXA-10, OXA-2 and OXA-46 clades, which are highlighted in the tree. The collapsed clades are based on [28, 66] and distinguished by red edges, and the size of the bubbles correspond to the number of proteins in the collapsed clade. The full version of the tree is available in Additional file 8 in Newick format. **Figure S6.** Prediction of the tertiary structures of sulfonamide resistance proteins using I-TASSER server [67]. Color spectrum, from blue to red, corresponds to the detected secondary structure of the proteins based on the order of the amino acids. C-score scales the confidence of each predicted structure between −5 to 2. Sul1: c-score = 0.86, Sul2: c-score = 1.20, Sul3: c-score = 1.25, Sul4: c-score = 1.07. **Figure S7.** Sequence Alignments of sulfonamide resistance proteins and a sensitive DHPS with a crystal structure stored in the Protein Data Bank (PDB). The alignment was performed in UCSF Chimera [68] using the Muscle algorithm [69]. α-Helixes and β-strands are marked with yellow and green colors, respectively. α-Helixes are more preserved than the β-strands and coils. **Table S1.** Functional verification of the synthesized putative novel resistance genes. **Table S2.** Sampling site coordinates for RS_Pune_. (DOCX 1985 kb)

Additional file 2: List of known ARGs, categorized by different families of antibiotics, identified as gene cassettes in both samples. (XLSX 20 kb)

Additional file 3: List of putative novel ARGs. (XLSX 110 kb)

Additional file 4: List of previously not reported gene cassettes. (XLSX 325 kb)

Additional file 5: Relative abundance of mobile sulfonamide resistance genes (*sul1–4*) in metagenomic samples containing *sul4*. (XLSX 28 kb)

Additional file 6: List of metagenomic samples searched for *sul4* genes. (XLSX 376 kb)

Additional file 7: Protein sequences of the synthesized genes in fasta format. (TXT 1 kb)

Additional file 8: Phylogenetic tree of the identified OXA-variant gene cassettes and 289 known OXA-variants retrieved from the CARD database. (TXT 9 kb)

Additional file 9: Phylogenetic tree of DHPS proteins encoded by chromosomal genes and mobile sulfonamide resistance genes. (TXT 283 kb)

## Abbreviations

ARG: Antibiotic resistance gene
DHPP: 7,8-Dihydropterin pyrophosphate
DHPS: Dihydropteroate synthase enzyme
ESBL: Extended spectrum beta-lactamase resistance
IR: Inverted repeat
ISCR: Insertion sequence common region
LR: Long read
MIC: Minimal inhibitory concentration
ORF: Open reading frame
pABA: Para-aminoBenzoic acid
PETL: Patancheru Enviro Tech Ltd., an industrial waste water treatment plant
RC: Rolling circle transposition
RS_PETL_: River sediment collected near PETL
RS_Pune_: River sediment collected in Pune
SMRT: Single-molecule real-time
SR: Short read
