# Supplementary material for: Discovery of the fourth mobile sulfonamide resistance gene
Source: Microbiome. 2017 Dec 15;5:160. doi: 10.1186/s40168-017-0379-y (PMC5732528; doi:10.1186/s40168-017-0379-y)
Supplement: Supplementary file 1 — Figure S1. Predicted functions of open reading frames recovered by the chromosomal integron primer pair MRG284-MRG285 separated by samples. The results are based on known homologues in the CARD database. Figure S2. Predicted functions of open reading frames of the “clinical” and “environmental” integrons from the HS464-GCP2 amplicons separated by samples. The results are based on known homologues in the CARD database. Figure S3. Functional annotation of the open reading frames not previously reported in integrons. The results are based on known homologues in the NCBI protein database. Putative resistance genes are determined based on annotation in the NCBI database. Figure S4. Genetic arrangements of functionally verified resistance gene cassettes as identified by PCR amplification of the integrons. The synthesized gene cassettes are distinguished by thicker borders. Both synthesized OXA-2-like gene cassettes have the same arrangement. Figure S5. Collapsed phylogenetic tree of the identified OXA-variant gene cassettes and 289 known OXA-variants retrieved from the CARD database. The identified genes are described by Id numbers and located adjacent to OXA-10, OXA-2 and OXA-46 clades, which are highlighted in the tree. The collapsed clades are based on [28, 66] and distinguished by red edges, and the size of the bubbles correspond to the number of proteins in the collapsed clade. The full version of the tree is available in Additional file 8 in Newick format. Figure S6. Prediction of the tertiary structures of sulfonamide resistance proteins using I-TASSER server [67]. Color spectrum, from blue to red, corresponds to the detected secondary structure of the proteins based on the order of the amino acids. C-score scales the confidence of each predicted structure between −5 to 2. Sul1: c-score = 0.86, Sul2: c-score = 1.20, Sul3: c-score = 1.25, Sul4: c-score = 1.07. Figure S7. Sequence Alignments of sulfonamide resistance proteins and a sensitive DHPS with a crystal struc [file 40168_2017_379_MOESM1_ESM.docx]

**
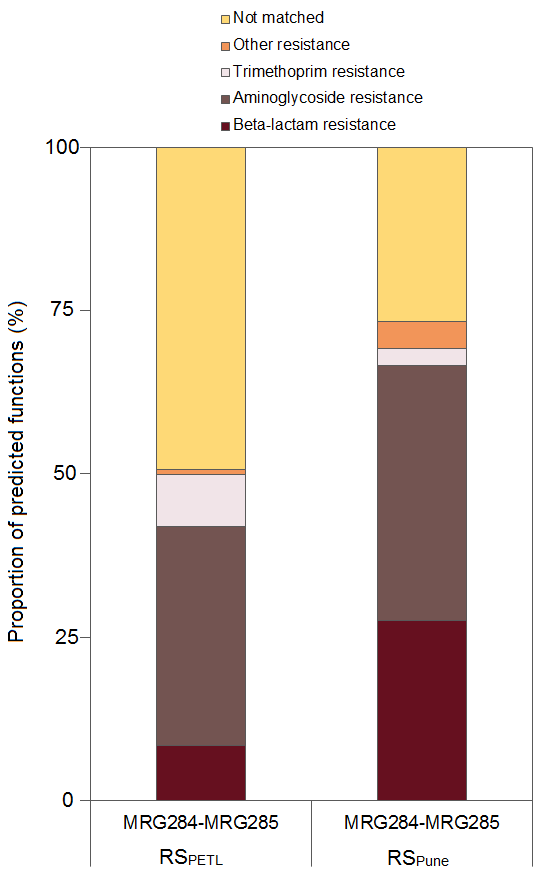
**

**Additional file 1:**

**Figure S1** Predicted functions of open reading frames recovered by the chromosomal integron primer pair MRG284-MRG285 separated by samples. The results are based on known homologues in the CARD database.


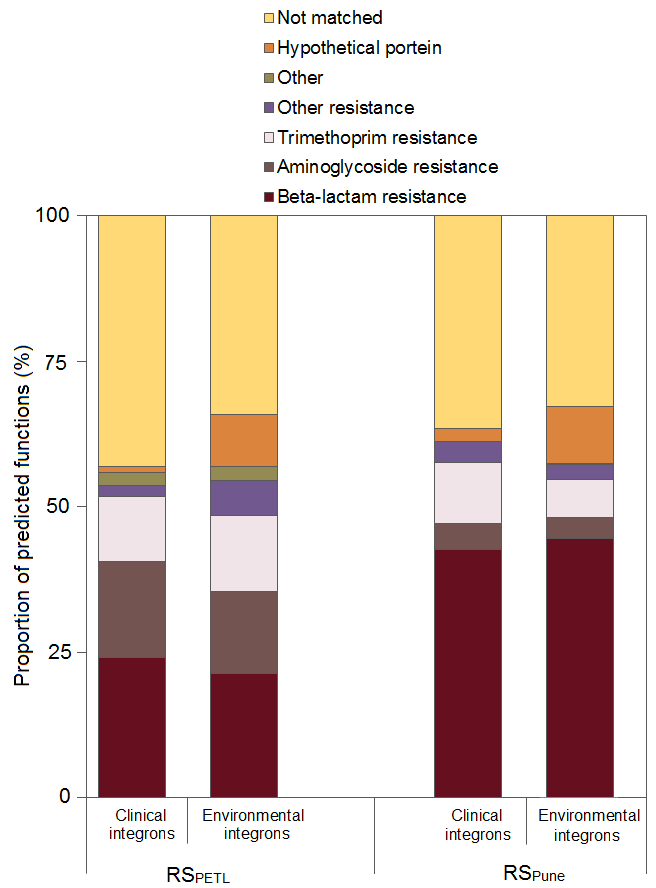


**Figure S2** Predicted functions of open reading frames of the “clinical” and “environmental” integrons from the HS464-GCP2 amplicons separated by samples. The results are based on known homologues in the CARD database.


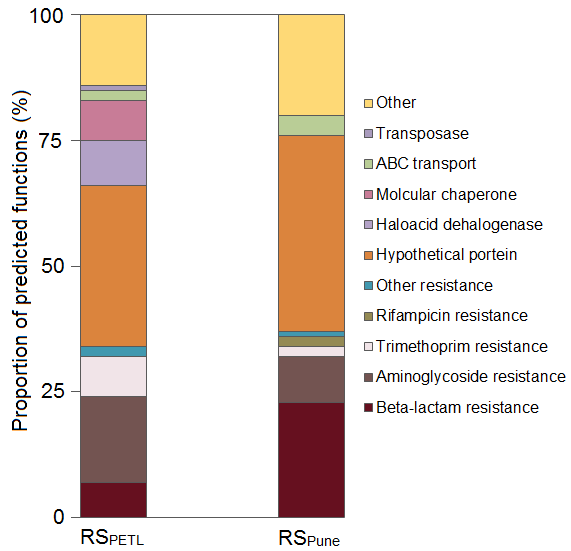


**Figure S3** Functional annotation of the open reading frames not previously reported in integrons. The results are based on known homologues in the NCBI protein database. Putative resistance genes are determined based on annotation in the NCBI database.


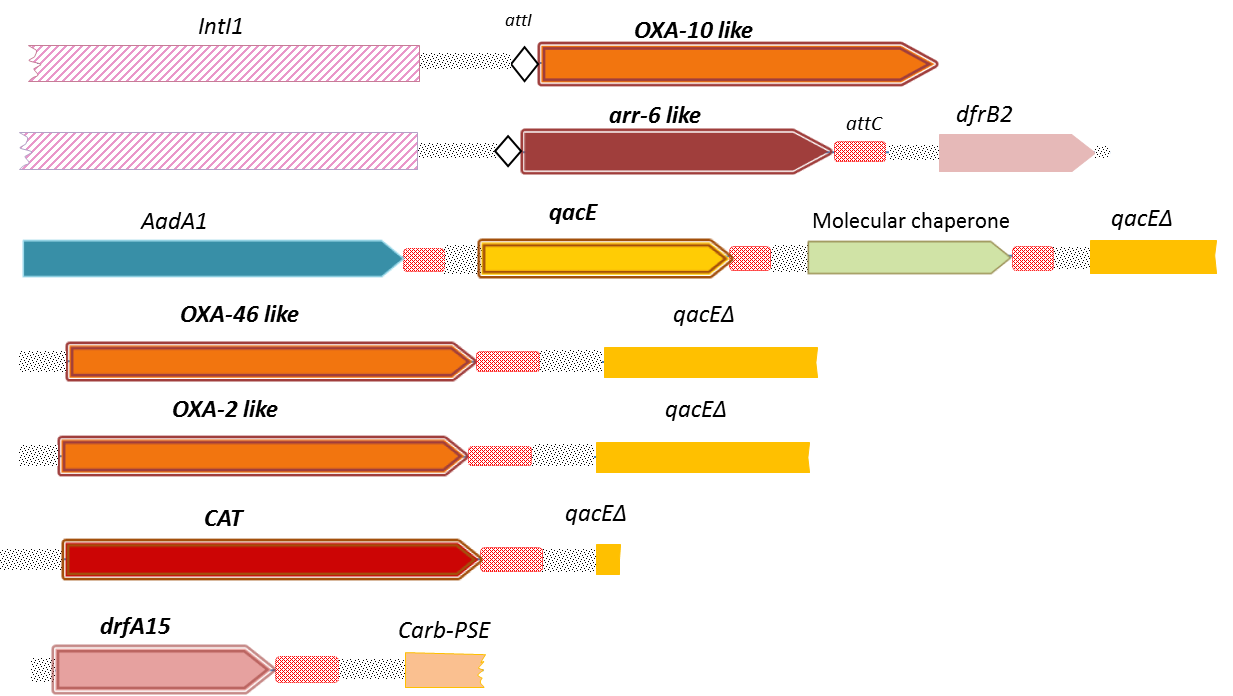


**Figure S4** Genetic arrangements of functionally verified resistance gene cassettes as identified by PCR amplification of the integrons. The synthesized gene cassettes are distinguished by thicker borders. Both synthesized OXA-2-like gene cassettes have the same arrangement.


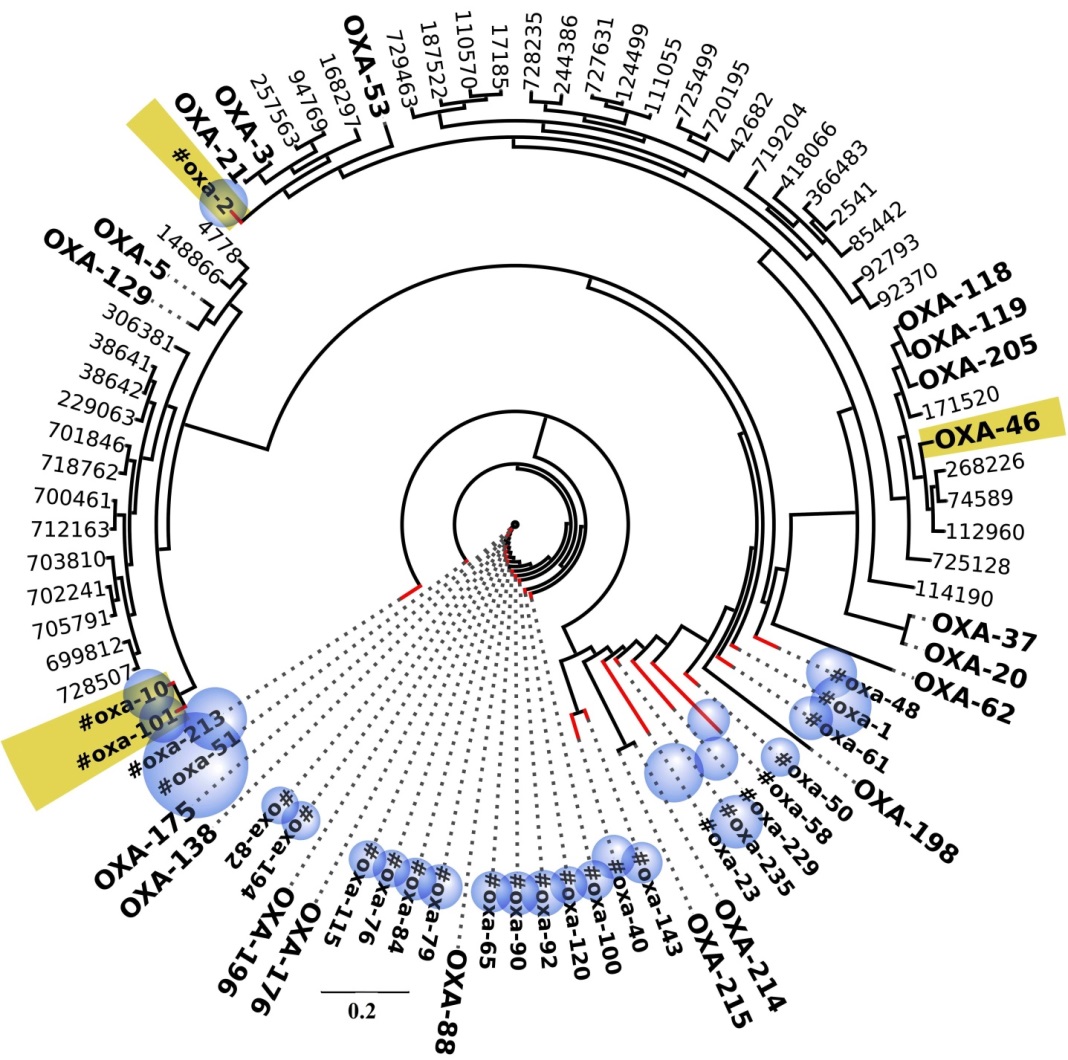


**Figure S5** Collapsed phylogenetic tree of the identified OXA-variant gene cassettes and 289 known OXA-variants retrieved from the CARD database. The identified genes are described by Id numbers and located adjacent to OXA-10, OXA-2 and OXA-46 clades, which are highlighted in the tree. The collapsed clades are based on [28, 67] and distinguished by red edges, and the size of the bubbles correspond to the number of proteins in the collapsed clade. The full version of the tree is available in Additional file 7 in Newick format.


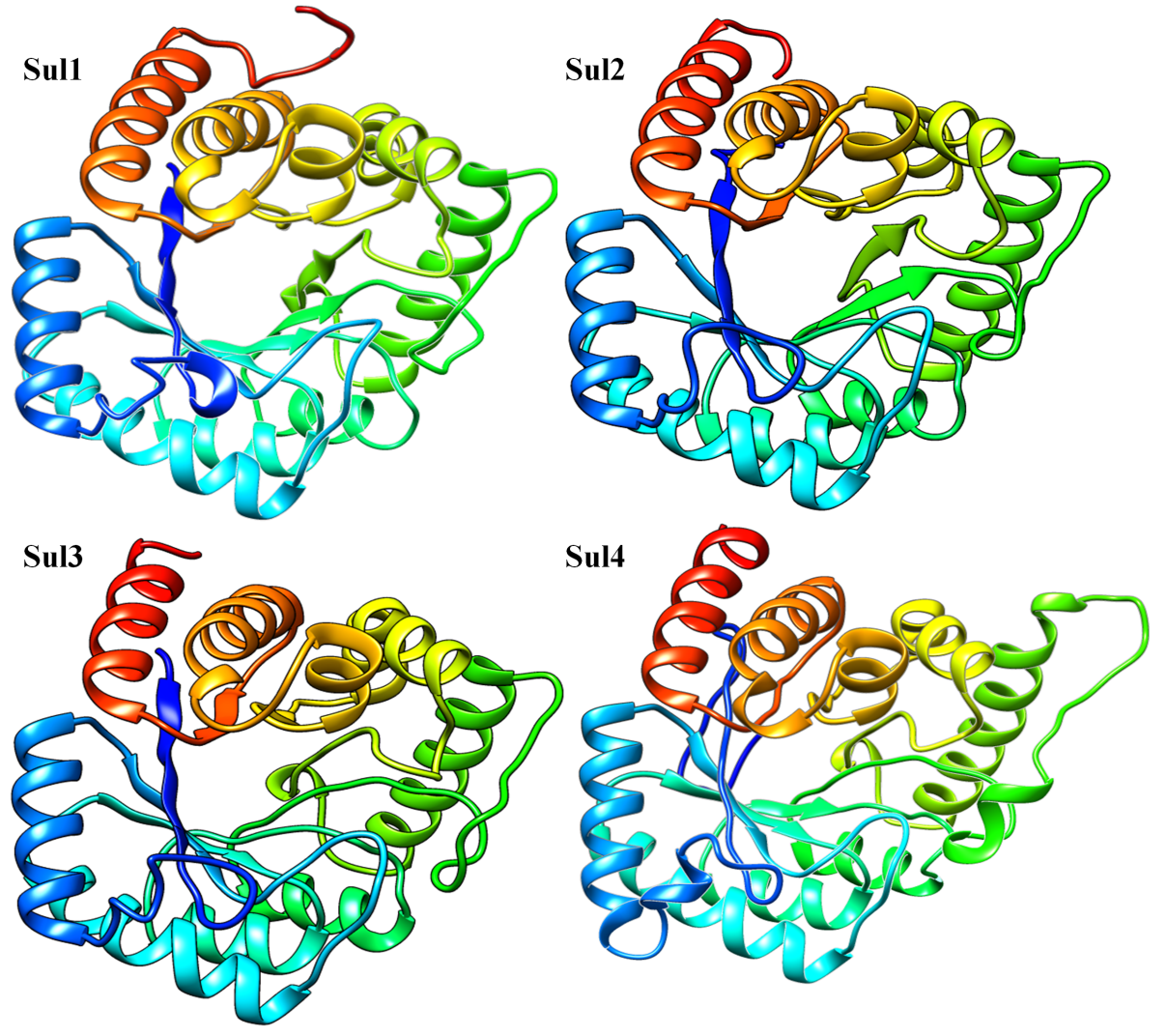


**Figure S6** Prediction of the tertiary structures of sulfonamide resistance proteins using I-TASSER server [68]. Colours spectrum, from blue to red, corresponds to the detected secondary structure of the proteins based on the order of the amino acids. C-score scales the confidence of each predicted structure between -5 to 2. Sul1: c-score=0.86, Sul2: c-score=1.20, Sul3: c-score=1.25, Sul4: c-score=1.07.


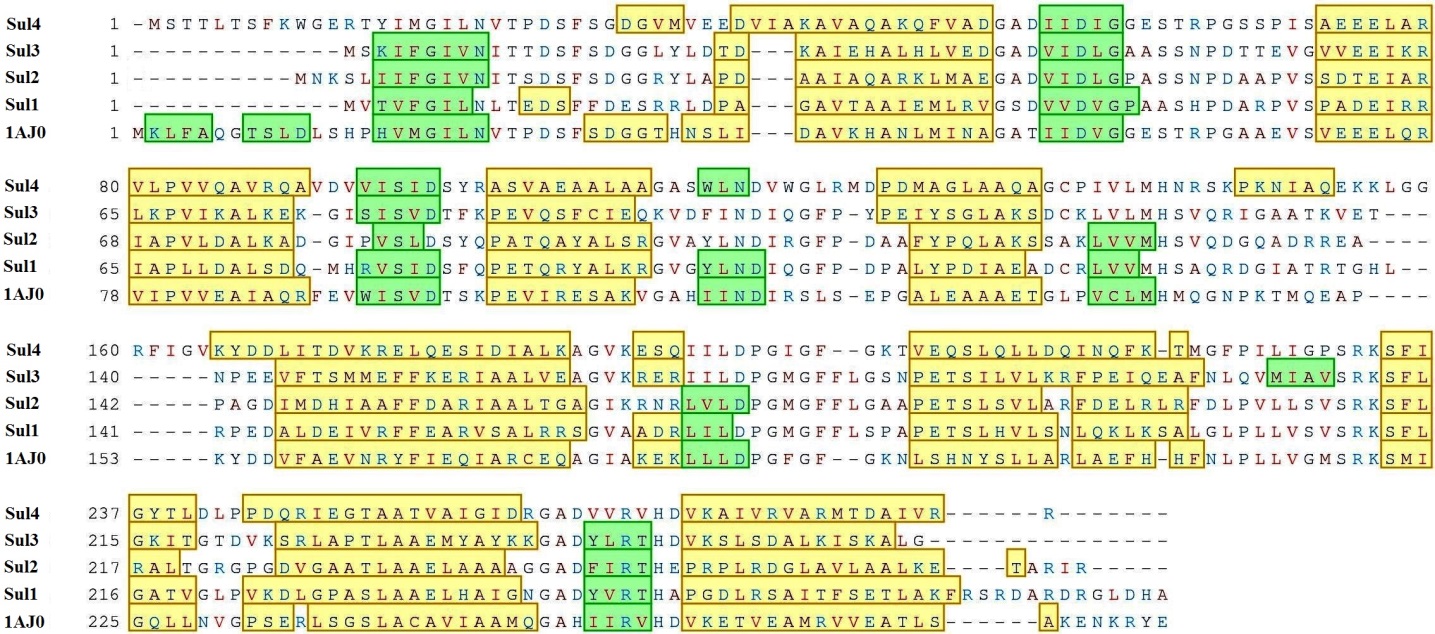


**Figure S7** Sequence Alignments of sulfonamide resistance proteins and a sensitive DHPS with a crystal structure stored in the Protein Data Bank (PDB). The alignment was performed in UCSF Chimera [69] using the Muscle algorithm [70]. α-Helixes and β-strands are marked with yellow and green colours, respectively. α-Helixes are more preserved than the β-strands and coils.

**Table S1** Functional verification of the synthesized putative novel resistance genes.

| **Sample** | **Amplicon Copy** | **Identity (%)** | **Coverage (%)** | **Description†** | **MIC (mg/l) value**  ***antibiotic*(*MIC*)(*Control*)** |
| --- | --- | --- | --- | --- | --- |
| Both | 62 | 69 | 100 | Dihydropteroate synthase | **SMX(>1024)(2)** |
| Both | 10 | 91 | 100 | OXA-2 | **AMP(>256)(4)**; **ETP(0.125)(0.006)**; IP(0.38)(0.19); CT(0.125)(0.047) |
| Both | 2 | 90 | 99.7 | OXA-2 | **AMP(>256)(4)**; **ETP(0.38)(0.006)**; IP(0.25)(0.19); CT(0.125)(0.047) |
| Both | 32 | 92 | 99.7 | OXA-10 | **AMP(>256)(4)**;  **CT(2)(0.047)**; **ETP(0.25)(0.006); IP(0.5)(0.19)**; |
| Both | 3 | 94 | 100 | Oxa-46 | **AMP(>256)(4)**; **ETP(0.25)(0.006)**; IP(0.38)(0.19); CT(0.125)(0.047) |
| RS_PETL_ | 3 | 92 | 99.6 | chloramphenicol O-acetyltransferase | **CHL(64)(3)** |
| Both | 174 | 81 | 100 | qacE | **SMX(6)(2)*** |
| RS_Pune_ | 1 | 88 | 100 | dfrA15 | **TMP(>32)(0.125)** |
| RS_PETL_ | 5 | 85 | 100 | Arr-6 | **RIF(>32)(4)** |

† Description of the closest hit in NCBI protein data base

*Not tested against quantium ammonium compound

SMX: sulphametoxazole; AMP: ampicillin; ETP: Ertapenem; IP: imipenem; CT: cefotaxime ; CHL: Chloramphenicol ; RIF: Rifampin; TMP: Trimethroprim

**Table S2** Sampling site coordinates for RS_Pune_.

| **Sampling site** | **Coordinates** | |
| --- | --- | --- |
| S3-Warje | 73°48’32.91’’ East | 18°28’27.54’’ North |
| S4-Deccan | 73°50’23.76’’ East | 18°30’37.30’’ North |
| S5-Bund garden | 73°53’29.62’’ East | 18°32’35.05’’ North |
